# Association between gene expression profile of the primary tumor and chemotherapy response of metastatic breast cancer

**DOI:** 10.1186/s12885-017-3691-9

**Published:** 2017-11-13

**Authors:** Cemile Dilara Savci-Heijink, Hans Halfwerk, Jan Koster, Marc Joan Van de Vijver

**Affiliations:** 1 Department of Pathology, Academic Medical Center, Meibergdreef 9, 1105 Amsterdam, AZ Netherlands; 2 Department of Oncogenomics, Academic Medical Center, Meibergdreef 9, 1105 Amsterdam, AZ Netherlands

**Keywords:** Adjuvant, Neoadjuvant, Chemosensitive, Chemoresistant

## Abstract

**Background:**

To better predict the likelihood of response to chemotherapy, we have conducted a study comparing the gene expression patterns of primary tumours with their corresponding response to systemic chemotherapy in the metastatic setting.

**Methods:**

mRNA expression profiles of breast carcinomas of patients that later developed distant metastases were analyzed using supervised and non-supervised classification techniques to identify predictors of response to chemotherapy. The top differentially expressed genes between the responders and non-responders were identified and further explored. An independent dataset which was generated to predict response to neo-adjuvant CT was utilized for the purpose of validation. Response to chemotherapy was also correlated to the clinicopathologic characteristics, molecular subtypes, metastatic behavior and survival outcomes.

**Results:**

Anthracycline containing regimens were the most common first line treatment (58.4%), followed by non-anthracycline/non-taxane containing (25.8%) and taxane containing (15.7%) regimens. Response was achieved in 41.6% of the patients to the first line CT and in 21.8% to second line CT. Response was not found to be significantly correlated to tumour type, grade, lymph node status, ER and PR status. Patients with HER2+ tumours showed better response to anthracycline containing therapy (*p*: 0.002). Response to first and second line chemotherapy did not differ among gene expression based molecular subtypes (*p*: 0.236 and *p*: 0.20). Using supervised classification, a 14 gene response classifier was identified. This 14-gene predictor could successfully predict the likelihood of better response to first and second line CT (*p*: <.0001 and *p*: 0.761, respectively) in the training set. However, the predictive value of this gene set in data of response to neoadjuvant chemotherapy could not be validated.

**Conclusions:**

To our knowledge, this is the first study revealing the relation between gene expression profiles of the primary tumours and their chemotherapy responsiveness in the metastatic setting. In contrast to the findings for neoadjuvant chemotherapy treatment, there was no association of molecular subtype with response to chemotherapy in the metastatic setting. Using supervised classification, we identified a classifier of chemotherapy response; however, we could not validate this classifier using neoadjuvant response data.

**Trial registration:**

Non applicable. Subjects were retrospectively registered.

**Electronic supplementary material:**

The online version of this article (10.1186/s12885-017-3691-9) contains supplementary material, which is available to authorized users.

## Background

The main aim of treating metastatic breast cancer is to prolong survival of the patients with acceptable toxicity and to palliate the disease-related symptoms. Response to combined chemotherapy agents varies between 50 and 70% in the metastatic setting [1, 2]. In order to avoid unnecessary chemotherapy treatment it would be of great benefit to be able to distinguish the group of patients which are not likely to respond to chemotherapy in general and to specific chemotherapy regimens. The decision to treat patients with metastatic breast cancer with chemotherapy is usually taken depending on many factors such as patient age and performance status, site of metastasis, hormone receptor status and prior exposure to chemotherapy, [3, 4]. Commonly used first-line therapeutic options in the metastatic setting include anthracycline- and/or taxane-based regimens [5]. In case of disease progression other cytotoxic agents may be applied to maximize the duration of quality time for these patients [6].

The current treatment approaches for metastatic disease consist to a large extent of trial-and-error type models, as predictors of response are lacking. The response rate to the chemotherapy regimens and the median duration of survival differs between breast cancer subtypes [7–11]. Several gene expression profiling studies aimed at the identification of a genomic predictor of chemotherapy response in the neoadjuvant setting have been performed and already provided important insights [12–17]. However a clinically validated gene expression profiling assay to predict the chemotherapy response has not yet been accomplished. Gene expression profiling studies of chemotherapy response in metastatic breast cancer have thus far been lacking.

We have previously investigated the association between the gene expression patterns of primary tumours and metastatic behavior in metastatic breast cancer [18].

In the current study, using the gene expression profiling data of 89 patients, the link between primary tumour and chemotherapy response in the frame of metastatic disease is explored. In order to develop genomic identifiers of chemotherapy responsiveness, gene expression patterns of the primary tumours of the responders and non-responders have been investigated.

## Methods

### Patients and tumour samples

Metastatic breast cancer patients from the *Academic Medical Center* and *Netherlands Cancer Institute (NCI)* were identified (*n* = 263) and a subgroup of patients from whom frozen tumour material from the primary tumour was available, were included in this study. This group constituted of 118 patients whose primary tumours were diagnosed between 1984 and 2000. The study protocol was approved by the Medical Ethical Committee of the *Academic Medical Center* and permission to use the data of the patients from *Netherlands Cancer Institute* was granted by the Core Facility-Molecular Pathology and Biobanking. Relevant clinical data and detailed information on metastatic behavior were abstracted from the clinical charts. Information related to metastatic behavior included data on site of metastasis (ever/never, first/not first and only/not-only for each metastasis site), metastasis pattern (uni/multiple) and metastasis timeline (early/late) has been previously published [11]. Time to develop metastatic disease, time from development of metastatic disease (metastasis specific survival, MSS) to last event and overall survival (OS) were recorded. Last event date was defined as the most recent follow up date for the patients who were alive and time of death for the others.

Histopathologic examination of the sections from the primary tumours was performed by two pathologists (C.D.S-H and M.J.V.) and as needed immunohistochemical stains and in-situ hybridization were applied in order to determine the hormone receptor and HER2 status as previously described [11].

#### Chemotherapy data

For each patient, administered systemic therapy was recorded for the adjuvant and metastatic settings separately. The therapy given was grouped as hormonal therapy (HT) and chemo-therapy (CT). In addition, the type of the therapeutic agent, the duration and the chronology of the therapy were noted. Due to the heterogeneity of the chemotherapy regimens, we have grouped the chemotherapy regimens into 3 groups as: anthracycline containing, taxane containing and non-anthracycline/non-taxane containing. Response to chemotherapy in metastatic patients was assessed for each line of chemotherapy according to RECIST [19] criteria and classified as complete response (CR), partial response (PR), stable disease (SD) and progressive disease. For statistical purposes, CR and PR were considered as response and SD and PD were considered as non-response. Response to first and second line CT and each chemotherapeutic group was separately assessed. Response to the given chemotherapy group were scored as response in case of response as first line treatment.

### Gene expression profiling

The gene expression profiling experiments have been described previously and detailed information on RNA amplification, labeling and hybridization can be found at Illumina website (https://www.illumina.com/) [18].The gene expression data was normalized utilizing robust spline normalization (rsn) and log2 transformed and followed by ComBat (https://www.bu.edu/jlab/wp-assets/ComBat/Abstract.html). Data analyses were conducted using R2 (Microarray Analysis and Visualization Platform), a publicly available web application (https://hgserver1.amc.nl/cgi-bin/r2/main.cgi).

For each tumour, the previously assessed 70-gene prognostic signature [20] was used to categorize tumours as good prognosis or poor prognosis signature. Genes were mapped to the Illumina platform via Gene Symbol ID. 62 Genes were found be present on the Illumina platform corresponding to 65 probes. The probe with the highest variance across the samples was selected in the event of existence of multiple probes for one gene. Tumours were assigned into the good or poor prognostic group based on the Pearson correlation coefficient between the centroids of the original good prognosis template and the gene expression levels of each sample. Classification into molecular subtypes (basal type, HER2 like, luminal A and luminal B type) were done using the genes from the PAM50 classifier [21]. The 21-gene recurrence score for each tumour was calculated as described by King et al. [22].

#### Identification and validation of predictors for chemotherapy response

To identify a gene expression predictor associated to response to chemotherapy, we used the one-way ANOVA function in R2 to select from a set of 15,526 genes with an expression level above background. 14 genes had a significant different expression (*p:* <0.001) between the group which the patient had a tumour response (CR and PR) to first line chemotherapy and the group in which the patient had no tumour response (SD and PD) to chemotherapy were identified. For validation, there are no published datasets for patients with metastatic disease; there are, however, various datasets of gene expression profiles of tumours from patients who underwent neoadjuvant chemotherapy treatment. Therefore, the predictive chemotherapy signature was then validated in an independent data set using the K-means and t-test function in R2. A data set (GSE25066) which includes 488 breast carcinomas with response data in the neoadjuvant setting was used for validation [12] .

To further investigate the association between this 14-gene predictor and clinical variables including response to chemotherapy multivariate logistic regression tests were applied using SPSS Statistics for Windows (Release version 21.0; IBM Corp.2012, Armond, NY). All statistical tests were two sided and *p <* 0.05 was considered to be statistically significant.

## Results

Gene expression profiles from primary tumours (*n* = 118) were assessed using microarrays. All patients were known to have developed distant metastasis and underwent (palliative) chemotherapy. The clinicopathologic features of the patients are displayed in Table 1. The mean age at diagnosis was 50.77 years (range 28 to 85 years). Median follow-up time was 63 months (range 9 to 211 months) for all patients and 136.50 months (range 74 to 208 months) for the patients who were alive at last follow-up. In this study group, 17.2% (*n* = 21) previously received neo-adjuvant systemic therapy and 80.4% (*n* = 98) adjuvant systemic therapy as part of the treatment of the primary tumour. Out of 98 patients who were given adjuvant therapy 39.8% (*n* = 39) received only chemotherapy, 15.3% (*n* = 15) received only hormonal therapy and 44.9% (*n* = 44) received chemotherapy and hormonal therapy. Adjuvant chemotherapy consisted of anthracycline containing regimens for 56.3%, non-anthracycline/non-taxane containing regimens for 42.3% and taxane containing drugs for 1.4% of the patients who received adjuvant chemotherapy (*n* = 71). None of the patients received trastuzumab as adjuvant treatment.

**Table 1:** Clinicopathologic characteristics of the primary tumours

		*N*	%
Age at diagnosis, years	<50	68	55,7%
	>50	54	44,3%
Histology	Ductal	105	86,8%
	Lobular	12	9,9%
	Other	4	3,3%
Tumour grade	1	9	7,6%
	2	69	58,5%
	3	40	33,9%
Lymph node status	none	35	30,2%
	1–3 positive	35	30,2%
	>3 positive	46	39,7%
Neoadjuvant chemotherapy	no	100	82,6%
	yes	21	17,4%
Adjuvant therapy	none	23	19,0%
	only CT	39	32,2%
	only HT	15	12,4%
	CT + HT	44	36,4%

Abbreviations: *CT* chemotherapy, *HT* hormonal therapy

In the metastatic setting all patients (*n* = 118) received palliative systemic therapy. Of these patients 49.2% (*n* = 58) received chemotherapy and hormonal therapy, 27.1% (*n* = 32) received only chemotherapy and 23.7% (*n* = 28) only hormonal therapy in the course of metastatic disease. The chemotherapeutic agents given in the metastatic setting were quite heterogeneous. As first line chemotherapy, 58.4% (*n* = 52) received an anthracycline containing regimen, 25.84% (*n* = 23) an non-anthracycline/non-taxane containing regimen and 15.73% (*n* = 14) received a taxane containing regimen (total *n* = 89). Second line CT was given to 63 patients and consisted of an anthracycline containing regimen for 22.2% (n = 14), a non-anthracycline/non-taxane containing regimen for 36.5% (n = 23) and a taxane containing regimen for 41.3% (*n* = 26) patients. Ten patients received a trastuzumab containing regimen as first line therapy.

The response rate for the first and second line chemotherapy was 41.6% and 21.8%, respectively. Patients who received anthracycline containing therapy showed a response rate of 51.8%, patients who received non-taxane/non-anthracycline containing therapy showed a response rate of 24.3% and the ones who were given taxane containing therapy had a response rate of 30.6%. Table 2 shows the distribution of the administered chemotherapy and response rates among patients. Response to chemotherapy was not found to be significantly correlated with histologic type, tumour grade and lymph node status. Response to first line chemotherapy treatment was better among patients who were younger than 50 years (*p*: 0.005).

**Table 2:** Distribution of the administered CT and response rates among patients

		Response	*N*	%
First line chemotherapy	A-CT	no	25	48,1%
		yes	27	51,9%
	NA/NT-CT	no	14	66,7%
		yes	7	33,3%
	T-CT	no	11	78,6%
		yes	3	21,4%
Second line chemotherapy	A-CT	no	10	71,4%
		yes	4	28,6%
	NA/NT-CT	no	20	87,0%
		yes	2	8,7%
	T-CT	no	17	68,0%
		yes	8	32,0%

Abbreviations: *A-CT* anthracycline containing chemotherapy, *NA/NT-CT* non-anthracyline/non-taxane containing chemotherapy, *T-CT* taxane containing chemotherapy

ER and PR status were not associated with response to chemotherapy treatment, whereas HER2 positive patients showed better response rate to anthracycline containing regimens (*p*: 0.002). Out of 13 HER2 positive patients with good response to anthracycline containing regimen, only 3 patients received trastuzumab for the treatment of metastatic disease (23.1%).

When classified into molecular subtypes 95 of the tumours classified as luminal (59, luminal A; 36, luminal B), 16 tumours as basal, 10 tumour as HER2-like and one tumour as normal like subtype. Out of luminal type tumours 65 and 49; of basal type tumours 16 and 8, of HER2-like tumours 9 and 5 received first and second line chemotherapy respectively. Response to first and second line chemotherapy did not differ among the molecular subtypes (*p*: 0.236 and *p*: 0.20). Molecular subtypes and their corresponding metastatic behavior have already been published [18].

Analyses were further carried out based on specific chemotherapy regimen. Anthracycline containing therapy was given to 41 patients with luminal type tumours, 7 patients with HER2-like tumours and 8 patients with basal type tumours as first or second line CT in the metastatic setting. Among these patients 51.21% of the patients with luminal type tumours, 71.4% of the patients with HER2-like tumours and 37.5% of the patients with basal type tumours showed response to anthracycline containing therapy (*p*: 0.624). Non-anthracycline/non-taxane containing therapy was given to 28 patients with luminal tumours, 3 patients with HER-2 type tumours and 6 patients with basal type tumours. Response rate to non-anthracycline/non-taxane containing regimens was 25%, 33.3% and 16.7% of the patients with luminal, HER2-type and basal type tumours, respectively (*p*: 0.954).

Taxane containing therapy was administered to 23 patients with luminal type tumours, 3 patients with HER2-type tumours and 10 patients with basal type tumours. Of luminal type tumours 39.1%, of HER2-type tumours 33.3% and of basal type tumours 10% responded to taxane containing therapy (*p*: 0.033). The association between the molecular subtypes of the tumours and their response status is displayed in Table 3.

**Table 3:** The association between the molecular subtypes of the primary tumours and chemotherapy response rates

	Response	Molecular subtype							
		Basal		Luminal A		Luminal B		HER2	
		*N*	%	*N*	%	*N*	%	*N*	%
A-CT	no	5	62,5%	12	48,0%	8	50,0%	2	28,6%
	yes	3	37,5%	13	52,0%	8	50,0%	5	71,4%
NA/NT-CT	no	5	83,3%	12	75,0%	9	75,0%	2	66,7%
	yes	1	16,7%	4	25,0%	3	25,0%	1	33,3%
				0		0		0	
T-CT	no	9	90,0%	4	36,4%	10	83,3%	2	66,7%
	yes	1	10,0%	7	63,6%	2	16,7%	1	33,3%

Abbreviations: *A-CT* anthracycline containing chemotherapy, *NA/NT-CT* non-anthracyline/non-taxane containing chemotherapy, *T-CT* taxane containing chemotherapy

The group of patients who received trastuzumab was composed of 6 with luminal type tumours, 3 with HER2-like tumours an 1 with a basal type tumour. There was no significant association between trastuzumab use and chemotherapy response (*p*: 0.291).

### Identification of genomic predictor(s) for chemotherapy response

**Fig. 1:**
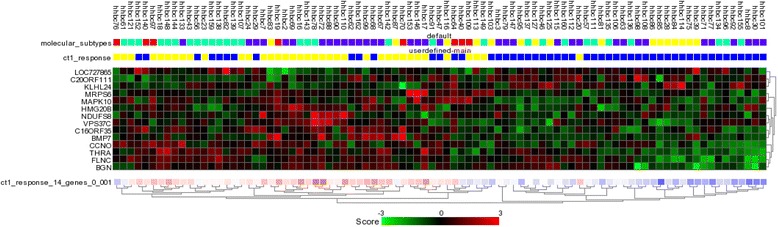
Heat map showing the gene expression pattern of 14-gene predictor for chemotherapy response. Heat map shows the gene expression profiling pattern of the 14 differentially expressed genes among 89 tumours. Primary tumours of the patients who respond to CT are illustrated in yellow and the ones without response are in blue. For each primary tumour the expression level of the specific gene is exhibited as red, if up-regulated and green, if down-regulated

Using supervised classification the differentially expressed genes between the primary tumours of metastatic breast cancer patients in responders (*n* = 37) and non-responders for the first line chemotherapy were explored (*n* = 52). Using supervised classification the top 14 differentially expressed genes between responders and non-responders were selected for further analyses. These 14 differentially expressed genes are listed as *BGN*, *BMP7*, *C16ORF35*, *C20ORF111*, *CCNO, FLNC, HMG20B*, *KLHL24*,, *LOC727865*, *MAPK10*, *MRPS6*, *NDUFS8*, *THRA* and *VPS37C*. Three of these genes were found to be down-regulated and the rest to be up-regulated in the group of patients with a good response to chemotherapy (Table 4). Figure 1 displays the expression profiling pattern of 14 differentially expressed genes among the patients. This heat map shows that the set of 14 genes separates the responders and non-responders in group of 89 tumours (*p*: < 0.001).

**Table 4:** Performance of the 14-gene predictor for chemotherapy response

			Training data set			Independent data set		
		Signature	Chemotherapy response					
			yes	no	*p*	yes	no	*p*
14-gene predictor ^a^	All	present	33	10	*2.24E-11*	47	158	*0.254*
		absent	4	42		52	231	
	ER-positive	present	23	2	*1.02E-10*	15	101	*0.327*
		absent	5	35		15	154	
	ER-negative	present	9	3	*3.37E-04*	41	63	*0.136*
		absent	0	12		27	66	

Abbreviations: *ER* estrogen receptor

^a^ The 14-gene predictor developed in this study

The correlation between these 14 differentially expressed genes and chemotherapy response was further explored. In the group of patients who received chemotherapy in the metastatic setting, 43 patients had a tumour with a “chemotherapy responsive” gene expression profile. Out of those 43 patients, 76.7% (*n* = 33) showed good response to first line therapy; whereas out of 46 patients who were predicted to be non-responsive to chemotherapy 91.3% had indeed no response (*p*: < .0001, sensitivity: 89.2%and specificity: 80.8%) In the case of response to second line CT, 37 were predicted to have good response with the 14-gene predictor and 24.3%(*n* = 9) of these showed good response; out of 27 tumours which were predicted as non-responder 81.5%(*n* = 22) had no response to CT (*p*: 0.249, sensitivity:64.3% and specificity: 44%). However, as this was the set of tumours in which the chemotherapy response signature was identified, validation in an independent dataset is required.

No other gene data set with chemotherapy response data in the metastatic setting was available for the validation of this gene set. Therefore an independent dataset with available chemotherapy response data for neo-adjuvant administered chemotherapy was utilized [12]. This data set included total 488 tumours with available information on chemotherapy response and 205 of these were predicted as responsive with the 14-gene predictor. Of these 205 tumours which were predicted as responsive, 47 (22.9%) showed response to chemotherapy. Out of 283 tumours which were assessed as non-responsive with the predictor, 231 (81.6%) had actually no response to CT (*p*:0.254, sensitivity: 47.5% and specificity: 59.4%). The validation of this 14-gene predictor is summarized in Table 4.

Other signatures which were developed to predict the response to neoadjuvant CT were also tested in this study group. DLDA30 signature correctly predicted 76.3% of the responsive and 62% of the non-responsive tumours (*p*: 6,5E-01). In contrast, the genomic grade index (GG1) and genomic predictor of Hatzis et al. were not able to distinguish the responsive and non-responsive groups (*p*: 0.317 and *p*: 0.212, respectively). The relationship between the 21-gene recurrence score and CT response in our study set was also further investigated in the subgroup of ER-positive/HER2-negative tumours. Out of 74 tumours 40.5% (*n* = 30) had low-risk, 16.2% (*n* = 12) had intermediate-risk and 43.2% (*n* = 32) had high- risk recurrence scores. A high-risk recurrence scores was found to be correlated with shorter overall survival time and time to develop metastases (*p*: 0.016 and p: 0.033, respectively); but not correlated with survival time after the development of metastatic disease (*p*: 0.117). The recurrence scores were not found to be correlated to chemotherapy response (*p*: 0.854).

Additional analyses to explore the correlation of the 14-gene predictor to the site of metastasis (bone metastasis ever, visceral metastasis ever, bone only metastasis and visceral only metastasis) have not revealed any significant relation (*p*: 0.72, *p*:0.58, *p*:0.38 and *p*:0.80, respectively). Yet it was found that this 14-gene predictor was significantly correlated to time to metastasis (metastasis within 5 year vs later than 5 year), more specifically tumours with present 14-gene expression profile developing metastases at a later time than the others (*p*: 0.021).

Survival analyses revealed no significant association between survival time (overall and metastasis specific) and chemotherapy responsiveness. Survival time also did not differ between patients with a responsive 14-gene predictor and the ones without it.

## Discussion

With the purpose of identifying a genomic predictor for response to chemotherapy in metastatic breast cancer, we have compared gene expression profiles of primary breast carcinomas to their response to chemotherapy treatment. We have identified a 14 gene expression profile associated with response to chemotherapy. This gene set was able to successfully predict the group of primary tumours which were more likely to respond to chemotherapy in the training set. We do not have access to a validation cohort of tumours from patients with metastatic breast cancer; therefore, we have studied the predictive value of the 14 gene predictive profile in published series of tumours from patients who underwent neoadjuvant chemotherapy treatment.

Specifically, Hatzis et al. have introduced a predictive test for neoadjuvant chemotherapy among patients with HER2-negative tumours. The chemopredictive test algorithm developed by this study was shown to predict the chemosensitivity with positive predictive value of 56% (95% CI, 31%–78%) and absolute risk reduction of 18% (95% CI, 6%–28%). When compared to the other predictive signatures such as genomic grade index (GG1), PAM50 and DLDA30 [13, 14, 21], the predictive algorithm of Hatzis et al. had greater positive predictive value in a validation cohort.

Neoadjuvant chemotherapy is increasingly employed for the treatment of breast cancer and predictors of response to neoadjuvant chemotherapy has been previously studied by several groups. Especially triple negative breast cancer (TNBC), which is characterized by lacking expression of ER, PR and HER-2, has shown to be more sensitive to systemic chemotherapy compared to the non-TNBC group. In particular, pathologic complete remission (pCR) has been reported to be achieved in 21.6–45% of TNBC patients. In contrast, hormone receptor positive tumours have been shown to be associated with very low pCR rates (4.9% -11%) [23–27]. Treatment of patients with HER2-positive tumours with chemotherapy plus HER2 targeted neoadjuvant therapy results in pCR rates of approximately 65% with 37% relative improvement in overall survival and an increase in 10-year overall survival rate from 75.2% to 84% [28–31]. Gene expression based analyses have shown similar results with basal like and HER2-type tumours having better pCR response to neoadjuvant chemotherapy (41.7% - 48.8%), compared to luminal type tumours which have shown to have response rates ranging from 2% to 8.2% [26, 32]. It is also known that, regardless of hormone receptor status and intrinsic subtype of the tumour, patients with residual disease after neoadjuvant chemotherapy have significantly shorter overall and disease free survival than patients who achieve pCR [23–25]. In this study identified chemotherapy response rates in the metastatic setting and their association with molecular subtypes and hormone receptor status differed from the ones in the neoadjuvant setting. Response rates to first line therapy given for metastatic disease was not found to be significantly different between molecular subtypes, i.e. basal like tumours and HER2-type tumours did not show better response rates compared to the luminal type tumours. On the other hand, HER-2 positive tumours were associated with better response which is in agreement with published studies [33, 34].

Recently, the Translational Breast Cancer Research Consortium (TBCRC) has conducted a study to explore the usefulness of the 21-gene recurrence score (RS) in predicting response to therapy among breast cancer patients presenting with Stage IV disease [22]. In the group of 69 patients with ER-positive/HER2-negative tumours, they have found that both time to first progression (TTP) and 2 year overall survival (OS) time were shorter for the patients with high-risk RS values (≥ 31) and who received first line endocrine therapy. There were no differences by means of TTP and 2-year OS in the group of patients with similarly high-risk RS values who received first-line chemotherapy. Therefore, the 21-gene RS has been suggested as a tool for selection of the patients presenting with stage IV ER-positive/HER2-negative breast cancer who may benefit from first-line chemotherapy. In the current study we have shown that ER-positive/HER2-negative tumours with high-risk recurrence scores had shorter time to develop metastatic disease and shorter overall survival, however we were not able to confirm an association with chemotherapy response.

In this study several limitations have been recognized. As already mentioned, heterogeneity of the given chemotherapeutic agents and non-availability of an independent gene expression data set with CT response information in the metastatic setting are the main limitations to be acknowledged. Nonetheless, the detailed information on response to CT in the setting of metastatic disease in a group of 118 patients is one of the strengths of this study.

## Conclusions

We present a comprehensive study comparing the gene expression patterns of primary tumours from metastatic breast cancer patients according to their responsiveness of chemotherapy during their treatment of metastatic disease. The 14 differentially expressed genes among these two groups have been further investigated and led to the exploration of couple genes that might play role in the response to CT. In contrast to the findings for neoadjuvant chemotherapy treatment, there was no association of molecular subtype with response to chemotherapy in the metastatic setting. Using supervised classification, we identified a classifier of chemotherapy response; however, we could not validate this classifier using neoadjuvant response data. We believe that the data generated in this study may inspire new studies leading to development of improved and individualized therapy strategies in treatment of metastatic breast cancer.

## Additional file

Additional file 1: Illumina microarray data generated in this study. (XLSX 160281 kb)

## Abbreviations

CT: Chemotherapy
ER: Estrogen receptor
HER2: Human epidermal growth factor receptor 2
HT: Hormonal therapy
MBC: Metastatic breast cancer
MSS: Metastasis specific survival
OS: Overall survival
PR: Progesterone receptor
RECIST: Response evaluation criteria in solid tumours
